# Maternal Consumption of Low-Isoflavone Soy Protein Isolate Confers the Increased Predisposition to Alcoholic Liver Injury in Adult Rat Offspring

**DOI:** 10.3390/nu10030332

**Published:** 2018-03-10

**Authors:** Sae Bom Won, Young Hye Kwon

**Affiliations:** 1 Department of Food and Nutrition, Seoul National University, Seoul 08826, Korea; newspring@snu.ac.kr; 2 Research Institute of Human Ecology, Seoul National University, Seoul 08826, Korea

**Keywords:** alcoholic liver disease, HDL cholesterol, one-carbon metabolism, rat offspring, soy protein isolate

## Abstract

Offspring of female rats fed either a casein (CAS) diet or a low-isoflavone soy protein isolate (SPI) diet were compared in an animal model of chronic ethanol consumption to investigate whether maternal diet regulates the adaptive responses of offspring to postnatal ethanol exposure and potentially affects the development of liver disease in later life. Female rats were fed either a CAS or an SPI diet before mating, and during pregnancy and lactation. Male offspring from the same litter were pair-fed either a control or ethanol diet for six weeks (CAS/CON, CAS/EtOH, SPI/CON, and SPI/EtOH groups). Serum aminotransferase activities and hepatic inflammatory indicators were higher in the SPI/EtOH group than in the CAS/EtOH group. Ethanol consumption increased serum homocysteine levels, hepatic *S*-adenosylmethionine:*S*-adenosylhomocysteine ratio, and hepatic endoplasmic reticulum stress only in offspring of SPI-fed female rats. Total and high-density lipoprotein (HDL) cholesterol levels and mRNA levels of hepatic genes involved in HDL cholesterol assembly were reduced in the SPI group in response to ethanol consumption. In conclusion, offspring of SPI-fed female rats were more susceptible to the later development of alcoholic liver disease than offspring of CAS-fed female rats. Furthermore, maternal SPI consumption altered one-carbon metabolism and cholesterol metabolism of offspring fed an ethanol diet.

## 1. Introduction

It was recently proposed that nutrition during the fetal and neonatal periods can affect growth and development as well as lifespan in mammals [1]. During mammalian development, the liver becomes a central metabolic organ that is responsible for metabolic homeostasis and detoxification [2]. Because the fetal liver is one of most vulnerable targets for extracellular signals that regulate cell proliferation, differentiation, and apoptosis, the programming of liver growth has a long-lasting effect on maintaining metabolic homeostasis in later health [3]. Indeed, the growth of liver is greatly decreased compared to other key organs, including brain and heart under poor maternal nutrition status [4].

There is growing evidence that the intrauterine environment can program adult disease susceptibility by epigenetic regulation, which results in altered chromatin structure and function [5,6]. Previously, we investigated whether maternal consumption of a low-isoflavone soy protein isolate (SPI) diet or a casein (CAS) diet with genistein regulates liver development in rat offspring [7]. SPI intake of dams—female rats that produced litters—resulted in higher cell proliferation and relative liver weight in offspring compared to CAS intake. Furthermore, maternal SPI consumption was associated with changes in gene expression, histone modification, and global and gene-specific DNA methylation patterns in livers from postnatal day 21 male offspring. An SPI diet also had a more profound effect on the lipid metabolism of their offspring compared to maternal consumption of a CAS diet with genistein [8]. These results imply that protein source may be a potential determinant of early liver development.

Chronic ethanol exposure is linked to liver injury, including fatty liver, hepatocyte necrosis, inflammation, fibrosis, and cirrhosis [9]. Among several alcohol-induced metabolic abnormalities, a disturbed metabolism of methionine and *S*-adenosylmethionine (SAM) is well documented [10]. Increased hepatic *S*-adenosylhomocysteine (SAH) accumulation by alcohol consumption leads to sensitization to tumor necrosis factor (TNF)-mediated hepatotoxicity [11]. In addition, altered one-carbon metabolism and endoplasmic reticulum (ER) stress in response to ethanol intake contribute to dysregulated lipid metabolism [12].

Although several studies have reported that maternal consumption of soy influenced the development and progression of diseases [13,14], few studies have shown the adaptive responses of offspring of dams fed a soy-containing diet to metabolic challenges. In the present study, offspring of dams fed either a CAS or an SPI diet were compared in an animal model of chronic ethanol consumption to investigate whether the adaptive responses of the offspring to environmental factors potentially affect the development of liver disease in later life. The role of one-carbon metabolism in alcoholic liver disease was also assessed.

## 2. Materials and Methods

### 2.1. Animals and Diets

**Figure 1:**
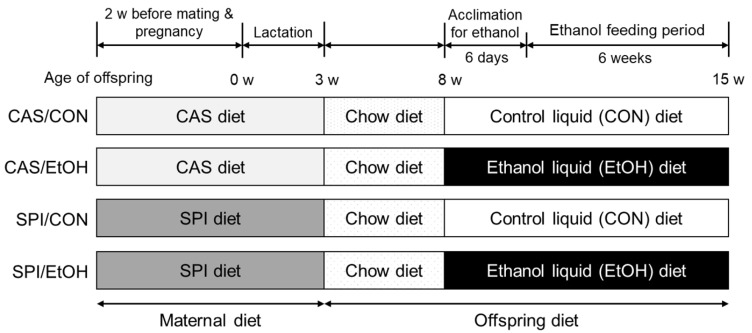
Overview of the study design. CAS, casein; SPI, low-isoflavone soy protein isolate.

The experimental diets and animal maintenance protocols used in this study have been previously described [7]. Briefly, 7 week-old virgin female Sprague—Dawley rats were obtained from the local animal facility (Orient Bio Inc., Seongnam, Korea). After 1 week of acclimation, the rats were randomly assigned to one of two experimental diets, a CAS diet and an SPI diet (7.47 mg of genistein, 29.42 mg of genistin, 2.73 mg of daidzein, and 12.81 mg of daidzin/kg diet). The experimental diets were made according to the published AIN-93G formula [15], except that soybean oil was replaced with corn oil. Female rats were fed a CAS diet or an SPI diet for 2 weeks before mating as well as throughout pregnancy and lactation. The male offspring were weaned to standard chow diet on postnatal day 21. At 8 weeks of age, pairs of offspring were randomly assigned to either control or ethanol group, resulting in a 2 × 2 study design. Two different ethanol groups (CAS/EtOH and SPI/EtOH) were fed a liquid ethanol Lieber–DeCarli diet (#710260; Dyets Inc., Bethlehem, PA, USA) containing 35% of the calories from fat, 11% from carbohydrate, 18% from protein, and 36% from ethanol [16]. Pair-fed control groups (CAS/CON and SPI/CON) were fed an ethanol-free isocaloric diet (#710027; Dyets Inc., Bethlehem, PA, USA) containing maltodextrin instead of ethanol. During an ethanol acclimation period, ethanol was gradually introduced into the liquid diet as 0%, 12%, and 24% of calories consumed for two days each. Thereafter, rats were maintained on 36% ethanol-derived calories for another 6 weeks. Ethanol groups were fed a liquid diet ad libitum and their daily intake was monitored. Control groups were pair-fed such that their food intake was restricted to the amount of food that the ethanol group had during the previous 24 h. Weight gain was measured once a week. At the end of the experiment, the offspring were sacrificed after an overnight fast, and the blood samples were rapidly obtained by cardiac puncture. After collection, the serum was obtained by centrifugation at 3000 rpm for 20 min at 4 °C and stored at −80 °C until analyzed. The tissues were removed, snap-frozen immediately in liquid nitrogen, and stored at –80 °C until use. The experimental procedures used in the present study were approved by the Seoul National University Institutional Animal Care and Use Committee (SNU-090330-6). The rats were maintained in a temperature (22 ± 3 °C)- and humidity (50 ± 10%)-controlled room, with a 12 h-dark–light cycle. The study scheme is shown in Figure 1.

### 2.2. Serum and Hepatic Biochemical Analysis

Serum triglyceride, total cholesterol, high-density lipoprotein (HDL) cholesterol, glutamic-oxaloacetic transaminase (GOT), and glutamic-pyruvic transaminase (GPT) levels were measured by using a commercial kit (Asan Pharmaceutical Co., Seoul, Korea). Serum total bile acid levels were determined by using a colorimetric assay kit (Gentaur, San Jose, CA, USA) and serum monocyte chemoattractant protein-1 (MCP1) levels were determined by using an ELISA kit (R&D Systems, Inc., Minneapolis, MN, USA) The total serum homocysteine levels were determined using a high-performance liquid chromatography (HPLC: UltiMate™ 3000 HPLC system; Dionex, Sunnyvale, CA, USA) according to a previously described protocol [17]. Briefly, a serum sample containing 2.5 mmol/L acetylcysteine as an internal control was reduced with tri-*n*-butylphosphine, and the proteins were precipitated with 10% trichloroacetic acid. Thiols were derivatized with ammonium 7-fluorobenzo-2-oxa-1,3-diazole-4-sulfonate and separated by reversed-phase HPLC using a 5 μm C18 bead column (Phenomenex, Torrance, CA, USA). The fluorescent intensities were observed at an excitation wavelength of 385 nm and an emission wavelength of 515 nm. The mobile phase consisted of 0.1 mol/L acetate buffer containing 2% methanol (pH 4.0) was filtered through a 0.45 μm filter and was degassed under vacuum. The isocratic flow rate was 1 mL/min. To ensure standardization between the sample runs, calibration standards were interspersed at intervals during each run.

Total lipids were extracted from liver samples according to the method of Folch et al. [18]. Hepatic triglyceride and cholesterol concentrations were determined by enzymatic colorimetric methods using the same commercial kits used in the serum (Asan Pharmaceutical Co., Seoul, Korea).

### 2.3. Hepatic SAM and SAH analysis

Hepatic SAM and SAH levels were determined using HPLC (P680 HPLC system; Dionex, Sunnyvale, CA, USA) according to a previously described method [19]. Briefly, liver samples were homogenized in 0.4 mol/L perchloric acid and were centrifuged at 10,000× *g* for 20 min at 4 °C. The supernatants were passed through a 0.2 μm filter and were injected into an HPLC apparatus equipped with a 5 μm C18 bead column and a UV-VIS detector operating at 254 nm. The mobile phase for eluting the SAM and SAH consisted of 0.1 mol/L sodium acetate, 5 mol/L heptanesulfonic acid, and 4.2% acetonitrile, adjusted to pH 4.5 with acetic acid. The mobile phase was filtered and was degassed under vacuum. The isocratic flow rate was 1 mL/min. To ensure standardization between the sample runs, calibration standards were interspersed at intervals during each run.

### 2.4. Liver Histology

Formalin-fixed liver tissue was processed into 4 μm thick paraffin sections and stained with hematoxylin and eosin (H&E) for histological evaluation. Briefly, paraffin-embedded liver sections were deparaffinized in xylene and rehydrated using graded alcohol. The tissue sections were stained with Harris’ hematoxylin for 8 min, washed with 1% HCl in 70% alcohol, dipped in ammonia water 5 times, and rinsed with distilled water. The sections were then stained with eosin for 5–10 s, dehydrated in graded alcohol, cleared with xylene, and coverslipped.

### 2.5. Total RNA Isolation and Real-Time PCR Analysis

Total RNA was isolated from liver tissue using RNAiso-Plus (Takara Bio Inc., Shiga, Japan). cDNA was synthesized using 2 μg of total RNA with the Superscript™II Reverse Transcriptase (Invitrogen, Carlsbad, CA, USA). All amplification reactions were performed using a StepOne™ Real Time PCR System (Applied Biosystems, Foster City, CA, USA) according to the manufacturer’s protocol. Amplification reactions of selective genes were performed using SYBR^®^ Green PCR Master Mix (Applied Biosystems, Foster City, CA, USA). The primer sequences are described in Supplementary Table S1. The commercially available TaqMan™ Assay primers and probes (Applied Biosystems, Foster City, CA, USA) were also used for other selected genes as listed in Supplementary Table S2. Beta-actin (ACTB) was used as a reference gene. Relative gene expression levels were analyzed using the 2^−ΔΔ*C*t^ assay.

### 2.6. Semi-Quantitative PCR

According to a previous report [20], the synthesized cDNA was amplified using primers for X-box binding protein 1 (XBP1, forward: 5′-GAACCAGGAGTTAAGGACACGC-3′ and reverse: 5′-GGGGATCTCTAAGACTACAGGCT-3′) and ACTB (forward: 5′-CCAGGGTGTGATGGTGGGTA-3′ and reverse: 5′-TACGACCAGAGGCATACAGG-3′). PCR reaction of XBP1 and β-actin was performed under the following condition: initiation at 94 °C for 4 min and PCR cycling for 25 cycles and 20 cycles (denaturation at 94 °C for 30 s, annealing at 58 °C for 30 s, and extension at 72 °C for 1 min), respectively. XBP1 primers generate cDNA products of the unspliced and spliced XBP1 mRNA. The unspliced fragment was further digested by *PstI* (Promega, Madison, WI, USA), resulting in two short fragments.

### 2.7. Total Protein Extraction and Immunoblotting

Liver tissues were homogenized in ice-cold protein lysis buffer. After centrifugation for 30 min at 10,000× *g* at 4 °C, the protein content of the supernatant was determined with a protein assay kit. Equal amounts of protein were loaded into the lanes of an SDS-PAGE gel, separated, and blotted onto a PVDF membrane. After blocking with 5% bovine serum albumin, membranes were probed with an anti-phosphorylated eukaryotic initiation factor 2 alpha antibody (p-eIF2α; Cell signaling Technology, Danvers, MA, USA) and an anti-total eIF2α antibody (Cell signaling Technology, Danvers, MA, USA). The membranes were then incubated with an IgG-peroxidase-conjugated secondary antibody for chemiluminescent detection. The band intensities were quantified using Quantity One software (Bio-Rad, Hercules, CA, USA).

### 2.8. Statistical Analysis

The data were expressed as the mean ± standard error of the mean (SEM). Statistical analyses were performed using the SPSS software (Version 19.0, IBM SPSS Inc., Armonk, NY, USA), and the differences were considered statistically significant at *p* < 0.05. Before statistical analysis, non-normally distributed parameters were log-transformed to approximate a normal distribution. For all experiments, the results were analyzed by two-way ANOVA to examine the main effects (maternal diet and ethanol diet) and their interaction. Significant ANOVA results were followed by Duncan’s multiple range test to determine whether there were differences between the means of multiple groups. Correlations between two variables were determined by Pearson’s correlation coefficient.

## 3. Results

### 3.1. Effects of Maternal Diet on Body and Organ Weights in Ethanol-Fed Rat Offspring

Although body weight at three weeks of age was significantly lower in offspring of dams fed an SPI diet than those of dams fed a CAS diet, there was no significant difference in body weights at beginning and end points of ethanol consumption among the groups (Table 1). The effect of ethanol diet was observed in both relative weights of liver and epididymal adipose tissue. However, there were no differences in relative organ weights between two ethanol-fed groups. Dietary intakes were not significantly different among the groups (CAS/CON: 97.2 ± 3.3 g/day, CAS/EtOH: 97.2 ± 3.3 g/day, SPI/CON: 100.7 ± 3.7 g/day, SPI/EtOH: 100.5 ± 3.7 g/day).

### 3.2. Effects of Maternal Diet on Liver Injury in Ethanol-Fed Rat Offspring

**Figure 2:**
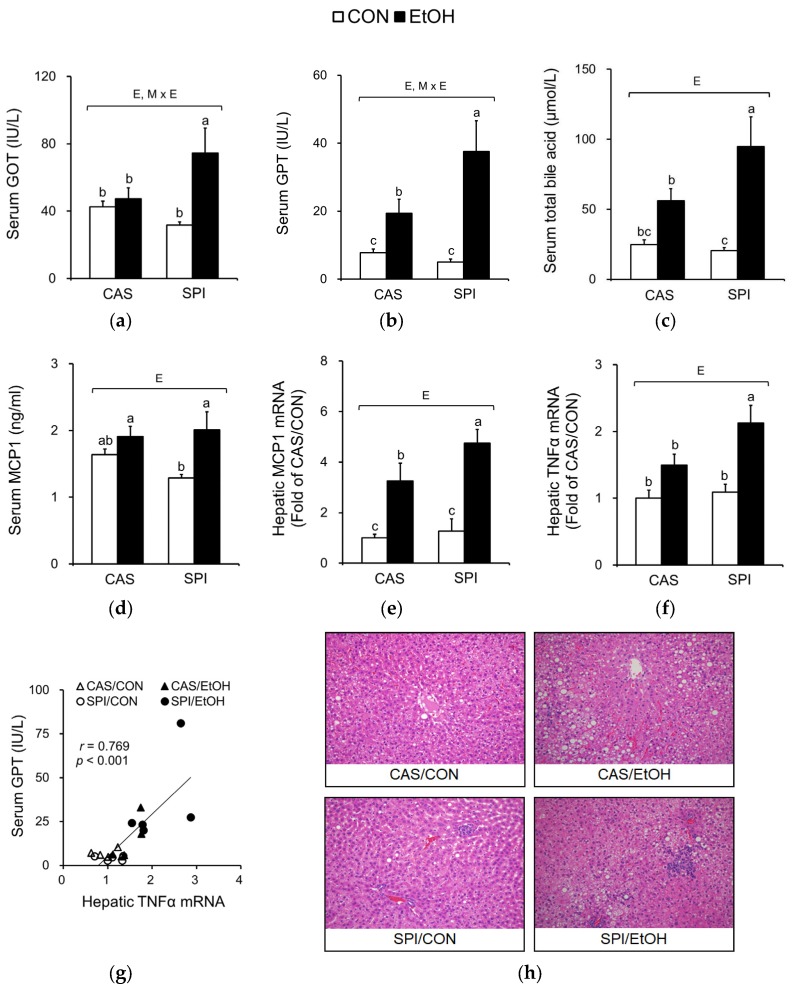
Effects of maternal diet on the liver injury in adult offspring fed an ethanol diet. (**a**) Serum glutamic-oxaloacetic transaminase (GOT) levels (*n* = 8), (**b**) serum glutamic-pyruvic transaminase (GPT) levels (*n* = 8), (**c**) serum bile acid levels (*n* = 8), (**d**) serum monocyte chemoattractant protein-1 (MCP1) levels (*n* = 7‒8), (**e**) hepatic MCP1 mRNA levels (*n* = 4‒5), (**f**) hepatic TNFα mRNA levels (*n* = 4‒5), (**g**) correlation between hepatic TNFα mRNA levels and serum GPT levels, and (**h**) representative H&E staining of liver tissue sections (*n* = 4). The mRNA levels were analyzed by real-time PCR and normalized to beta-actin (ACTB) as an endogenous control. Data are expressed as means ± SEM. Significant effects of maternal diet (M), offspring ethanol diet (E), and their interaction (M × E) were analyzed by two-way ANOVA (*p* < 0.05). Means without a common superscript significantly differ based on post-hoc analysis (*p* < 0.05). Pearson correlation coefficient, *r*, and *p*-value are indicated. Data that were not normally distributed were log-transformed before the statistical analysis. CAS, casein; SPI, low-isoflavone soy protein isolate; CON, control; EtOH, ethanol.

Hepatic injury markers, serum GOT and GPT activities and total bile acid levels were significantly higher in the SPI/EtOH group compared to the CAS/EtOH group (Figure 2a–c). The interaction effect of maternal diet and ethanol diet was observed in serum GOT and GPT activities. Serum MCP1 levels and hepatic gene expressions of MCP1, a chemokine that contributes to chronic alcoholic liver injury, were significantly increased in the SPI/EtOH group compared with the SPI/CON group (Figure 2d,e). Ethanol intake also significantly induced hepatic tumor necrosis factor alpha (TNFα) mRNA levels only in the SPI group (Figure 2f). Hepatic TNFα mRNA levels and serum GPT levels were significantly correlated, suggesting that hepatic inflammation is an important contributor to liver injury (Figure 2g).

Furthermore, representative images of H&E stained liver sections revealed higher infiltrated inflammatory cells in the SPI/EtOH group compared to the CAS/EtOH group (Figure 2h).

### 3.3. Effects of Maternal Diet on One-Carbon Metabolism in the Liver of Ethanol-Fed Offspring

**Figure 3:**
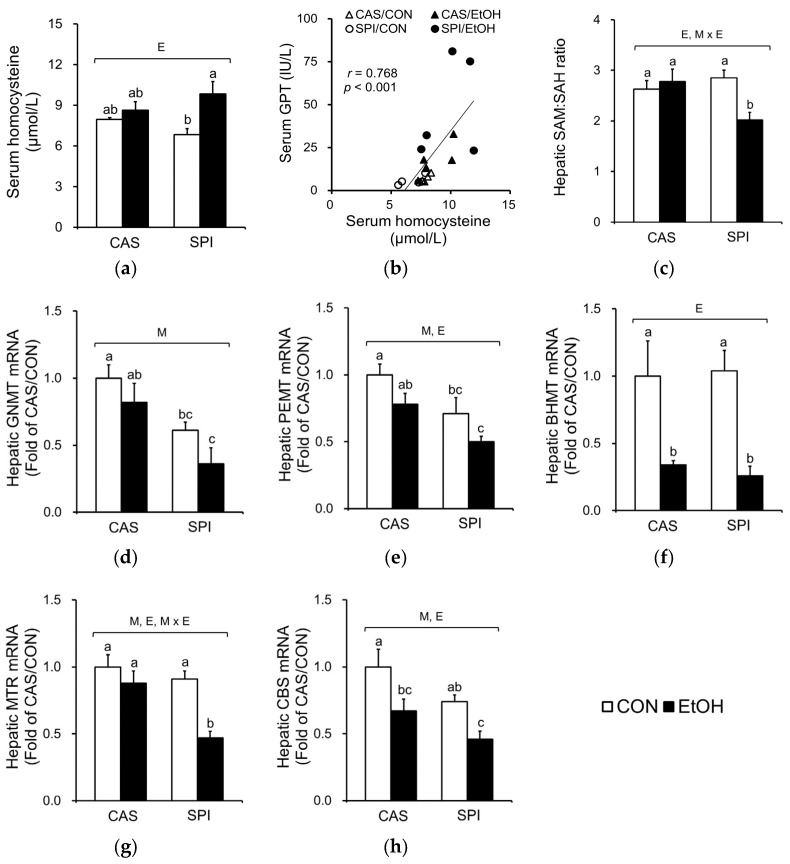
Effects of maternal diet on homocysteine and one-carbon metabolism in adult offspring fed an ethanol diet. (**a**) Serum homocysteine levels (*n* = 5) and (**b**) their correlation with serum GPT levels; (**c**) Hepatic *S*-adenosylmethionine (SAM):*S*-adenosylhomocysteine (SAH) ratio (*n* = 7‒8); Hepatic mRNA levels of genes involved in (**d**–**f**) methylation reaction and (**g**,**h**) homocysteine metabolism (*n* = 5). The mRNA levels were analyzed by real-time PCR and normalized to ACTB as an endogenous control. Data are expressed as means ± SEM. Significant effects of maternal diet (M), offspring ethanol diet (E), and their interaction (M × E) were analyzed by two-way ANOVA (*p* < 0.05). Means without a common superscript significantly differ based on post-hoc analysis (*p* < 0.05). Pearson correlation coefficient, *r*, and *p*-value are indicated. Data that were not normally distributed were log-transformed before the statistical analysis. CAS, casein; SPI, low-isoflavone soy protein isolate; CON, control; EtOH, ethanol.

One-carbon metabolism is comprised of folate and methionine cycles and supports the critical function of various methyltransferase reactions. Altered one-carbon metabolism is well documented in alcoholic liver disease, with an increased serum homocysteine concentration and a decreased hepatic SAM:SAH ratio. Ethanol ingestion significantly increased serum homocysteine levels only in the SPI group (Figure 3a). A significant correlation between serum homocysteine and GPT levels was observed (Figure 3b). Furthermore, we observed a significantly lower hepatic SAM:SAH ratio in the SPI/EtOH group compared to the CAS/EtOH group with a significant interaction effect (Figure 3c). Among the major methyltransferases that mediate the SAM-dependent transmethylation reactions, hepatic glycine *N*-methyltransferase (GNMT) and phosphatidylethanolamine *N*-methyltransferase (PEMT) mRNA levels were significantly lower in the SPI/EtOH group compared to the CAS/EtOH group (Figure 3d,e). Ethanol intake significantly reduced betaine-homocysteine *S*-methyltransferase (BHMT) mRNA levels in both CAS and SPI groups with no significant difference between two groups (Figure 3f).

We also determined mRNA levels of enzymes involved in remethylation of homocysteine (Figure 3g,h). The mRNA levels of 5-methyltetrahydrofolate-homocysteine methyltransferase (MTR) were significantly lower in the SPI/EtOH group compared to the CAS/EtOH group, suggesting that disturbed remethylation may contribute to higher serum homocysteine levels. Furthermore, maternal diet significantly regulated mRNA levels of cystathionine beta-synthase (CBS), a key regulatory enzyme in transsulfuration pathway.

Negative correlations between serum GPT levels and mRNA levels of genes involved in one-carbon metabolism are listed in Table 2.

### 3.4. Effects of Maternal Diet on ER Stress in the Liver of Ethanol-Fed Rat Offspring

**Figure 4:**
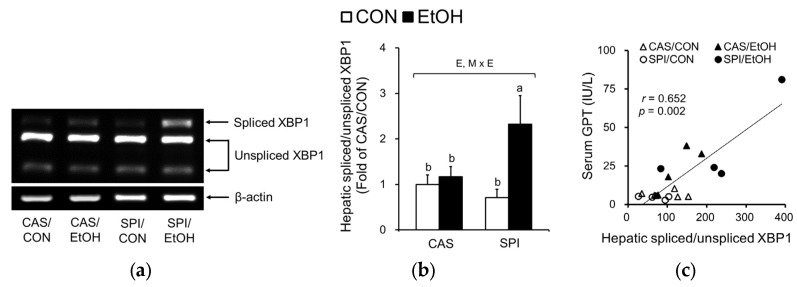

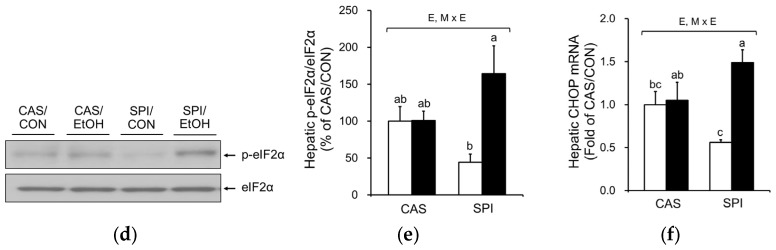
Effects of maternal diet on the endoplasmic reticulum (ER) stress response in adult offspring fed an ethanol diet. (**a**,**b**) Hepatic spliced:unspliced XBP1 mRNA levels were analyzed by semi-quantitative PCR and normalized to ACTB as an endogenous control (*n* = 4–5). (**c**) Correlation between hepatic spliced:unspliced XBP1 mRNA levels and serum GPT levels. (**d**,**e**) Hepatic p-eIF2α protein levels were analyzed by immunoblotting and normalized to total eIF2α protein levels. (**f**) Hepatic CCAAT/enhancer binding protein homologous protein (CHOP) mRNA levels were analyzed by real-time PCR and normalized to ACTB as an endogenous control (*n* = 5). Data are expressed as means ± SEM. Significant effects of maternal diet (M), offspring ethanol diet (E), and their interaction (M × E) were analyzed by two-way ANOVA (*p* < 0.05). Means without a common superscript significantly differ based on post-hoc analysis (*p* < 0.05). Pearson correlation coefficient, *r*, and *p*-value are indicated. Data that were not normally distributed were log-transformed before the statistical analysis. CAS, casein; SPI, low-isoflavone soy protein isolate; CON, control; EtOH, ethanol.

Hyperhomocysteinemia has been reported to induce ER stress in alcoholic liver injury [21,22]. Accordingly, we observed a significantly higher ER stress in the SPI/EtOH group compared to the SPI/CON group as observed in spliced form of XBP1 mRNA levels (Figure 4a,b), which were significantly correlated with serum GPT levels (Figure 4c). In addition, p-eIF2α levels were significantly increased in offspring of dams fed an SPI diet in response to ethanol (Figure 4d,e). CCAAT/enhancer binding protein homologous protein (CHOP) mRNA levels also showed a significant maternal diet and ethanol feeding interaction effect (Figure 4f).

### 3.5. Effects of Maternal Diet on Serum and Hepatic Lipid Profiles in Ethanol-Fed Rat Offspring

**Figure 5:**
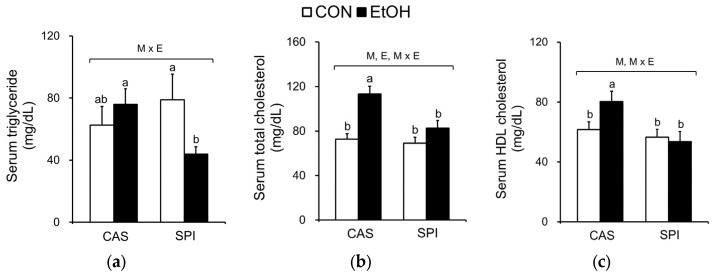

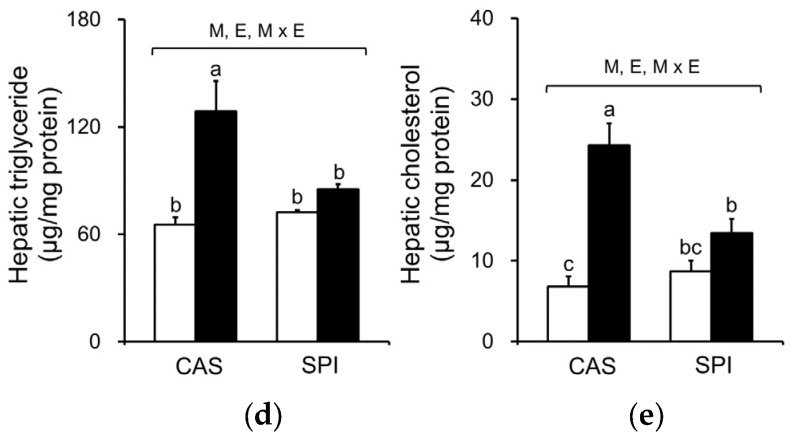
Effects of maternal diet on serum and hepatic lipid levels in adult offspring fed an ethanol diet. Serum (**a**) triglyceride, (**b**) total cholesterol, and (**c**) HDL cholesterol levels (*n* = 8); Hepatic (**d**) triglyceride and (**e**) cholesterol levels (*n* = 7‒8). Data are expressed as means ± SEM. Significant effects of maternal diet (M), offspring ethanol diet (E), and their interaction (M × E) were analyzed by two-way ANOVA (*p* < 0.05). Means without a common superscript significantly differ based on post-hoc analysis (*p* < 0.05). Data that were not normally distributed were log-transformed before the statistical analysis. CAS, casein; SPI, low-isoflavone soy protein isolate; CON, control; EtOH, ethanol.

Because ethanol consumption is associated with abnormal lipid metabolism, we investigated serum and hepatic lipid profiles in offspring of dams fed an SPI diet. As shown in Figure 5a–c, we observed that serum triglyceride, total cholesterol, and HDL cholesterol levels exhibited significant interaction effects between maternal diet and postnatal ethanol consumption. These parameters were significantly lower in the SPI/EtOH group compared to the CAS/EtOH group. Ethanol intake significantly increased hepatic triglyceride and cholesterol levels in offspring of dams fed a CAS diet, but not in offspring of dams fed an SPI diet (Figure 5d,e). Consistently, we observed an increased lipid accumulation in the CAS/EtOH group compared to the SPI/EtOH group (Figure 2h). Here, we observed a separate regulation of hepatic steatosis and inflammation in response to ethanol feeding. Previous studies have reported that hepatic triglyceride accumulation was protective against liver damage by reducing cellular oxidative stress and free fatty acids [23,24].

### 3.6. Effects of Maternal Diet on Serum and Hepatic Cholesterol Metabolism in Ethanol-Fed Rat Offspring

**Figure 6:**
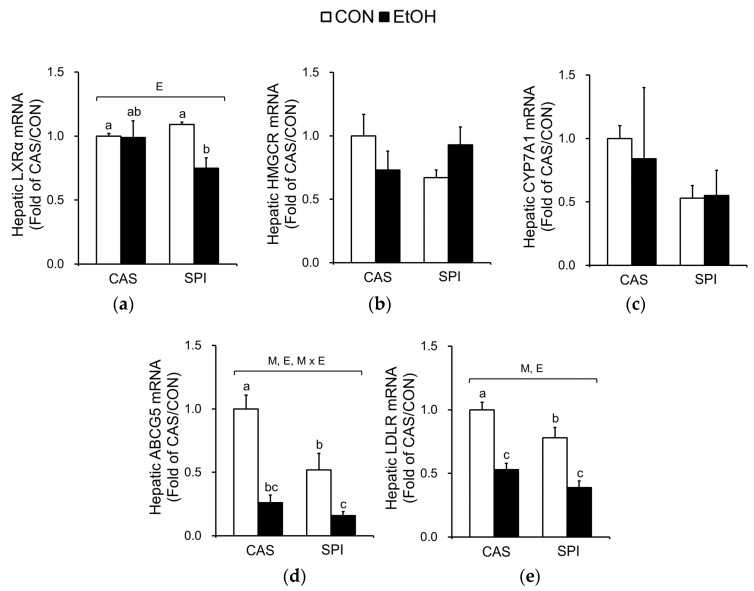

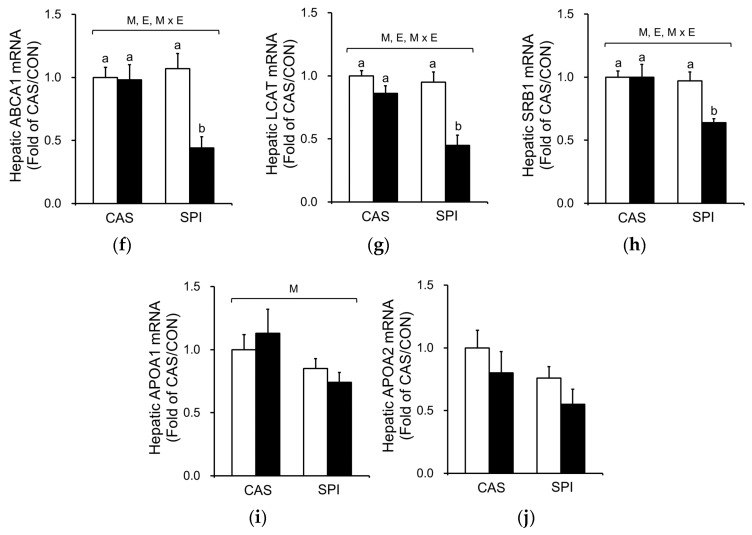
Effects of maternal diet on the gene expression related to cholesterol metabolism in the liver of adult offspring fed an ethanol diet. Hepatic mRNA levels of genes involved in (**a**–**e**) cholesterol metabolism and (**f**–**j**) HDL cholesterol metabolism (*n* = 4‒5). The mRNA levels were analyzed by real-time PCR and normalized to ACTB as an endogenous control. Data are expressed as means ± SEM. Significant effects of maternal diet (M), offspring ethanol diet (E), and their interaction (M × E) were analyzed by two-way ANOVA (*p* < 0.05). Means without a common superscript significantly differ based on post-hoc analysis (*p* < 0.05). Data that were not normally distributed were log-transformed before the statistical analysis. CAS, casein; SPI, low-isoflavone soy protein isolate; CON, control; EtOH, ethanol.

To determine the underlying mechanisms in altered cholesterol metabolism, we measured mRNA levels of key regulatory genes. The mRNA levels of liver X receptor alpha (LXRα), a gene encoding a major transcription factor involved in cholesterol metabolism, were significantly decreased only in the SPI group in response to ethanol intake (Figure 6a). Although mRNA levels of 3-hydroxy-3-methylglutaryl coenzyme A reductase (HMGCR), a gene encoding a rate-limiting enzyme in cholesterol biosynthesis, were not significantly different among the groups, they tended to have an interaction effect (*p* = 0.064) (Figure 6b). Expressions of cholesterol 7α hydroxylase (CYP7A1), a gene encoding a rate-limiting enzyme involved in bile synthesis, were not significantly different among the groups (Figure 6c). Ethanol intake significantly reduced mRNA levels of ATP-binding cassette subfamily G member 5 (ABCG5), a gene involved in biliary cholesterol secretion, and low density lipoprotein receptor (LDLR) in both CAS and SPI groups (Figure 6d,e).

The mRNA levels of genes involved in HDL cholesterol metabolism were also determined. ATP-binding cassette subfamily A member 1 (ABCA1) is involved in an initial assembly of HDL by transferring of cellular phospholipids and cholesterol to extracellular lipid-poor apolipoprotein A1 (APOA1) [25]. Lecithin cholesterol acyltransferase (LCAT) esterifies pre-HDL cholesterol, and this esterified cholesterol returns to the liver via scavenger receptor class B member 1 (SRB1). Gene expressions of ABCA1, LCAT, and SRB1 were significantly suppressed by ethanol intake and their mRNA levels in the SPI/EtOH group were significantly lower than in the CAS/EtOH group (Figure 6f–h). Both APOA1 and APOA2 mRNA levels tended to be lower in the SPI/EtOH group compared to the CAS/EtOH group, although the trend was not statistically significant (Figure 6i,j). These results suggest that altered HDL assembly resulted in lower serum total and HDL cholesterol levels in the SPI/EtOH group compared to the CAS/EtOH group.

Negative correlations between mRNA levels of genes involved in cholesterol metabolism and serum GPT levels are listed in Table 3.

## 4. Discussion

Several studies have suggested that soy components other than isoflavones may play important roles in influencing the susceptibility of offspring to the later development of certain diseases. Therefore, we investigated whether maternal diet with different protein sources, either casein or SPI, programs hepatic metabolic responses to chronic ethanol exposure in adult offspring. In the present study, ethanol intake increased serum homocysteine levels and altered hepatic one carbon metabolism in offspring of dams fed an SPI diet, but not in offspring of dams fed a CAS diet. Furthermore, increased inflammatory activities and altered cholesterol metabolism were observed in offspring of dams fed an SPI diet.

Changes in one-carbon metabolism have been shown to be associated with offspring growth, metabolism, and health [6]. We have recently identified that SPI consumption in dams apparently altered hepatic one-carbon metabolism in three-week old offspring [7]. Here, maternal dietary effect on one-carbon metabolism was also observed in adult offspring. In addition to a reduced hepatic SAM:SAH ratio, the SPI/EtOH group exhibited significantly lower PEMT and GNMT mRNA levels compared to the CAS/EtOH group. Similarly, a previous study has reported decreased mRNA levels for multiple methyltransferases in liver biopsies from patients with alcoholic hepatitis [21]. It is also noteworthy that *Pemt*−/− mice exhibit the hallmark of alcoholic liver injury, including increased fat deposition and apoptosis, and abnormal choline metabolism [26]. In *Gnmt*−/− mice, chronic hepatitis and glycogen storage diseases were shown to be developed [27]. Furthermore, severely affected strains of mice by chronic ethanol feeding exhibited hyperhomocysteinemia and major differences in the expression of genes involved in one-carbon metabolism and ER stress pathway [28]. Based on the above considerations, we propose that an impaired one-carbon metabolism contributes to a disruption of hepatic defense system in offspring of dams fed an SPI diet. Indeed, elevated SAH levels have been shown to sensitize hepatocytes to TNF-induced toxicity in alcoholic liver disease [21]. The severity of alcoholic liver injury was shown to be attenuated by SAM [29], betaine [30], and phosphatidylcholine [31] supplementation or in transgenic extrahepatic expressions of BHMT [22].

Serum lipid analysis may be useful as an indicator reflecting the pathologic conditions of liver [32]. Previous studies have reported that serum lipid levels are significantly decreased in liver diseases [33,34]. It is known that lipid and lipoprotein metabolism could be regulated by cytokines, which were shown to delay intestinal lipid absorption, inhibit LCAT activity, and decrease lipoprotein secretion [34]. Particularly, HDL-cholesterol level may be a useful index that evaluates the severity of liver diseases. Serum lipid profile, including total and HDL cholesterol, had a negative correlation with the severity of liver disease [35]. Here, we observed significantly lower levels of serum total and HDL cholesterol in the SPI/EtOH group compared to the CON/EtOH group. Consistently, expressions of target genes involved in HDL cholesterol assembly were strongly associated with serum GPT levels. In addition, several studies have reported that hyperhomocysteinemia is associated with a decrease in serum HDL cholesterol [36]. *Cbs*−/− mice were shown to have decreased LCAT mRNA levels [37] and *Mthfr*+/− mice were shown to have decreased APOA1 mRNA levels [38]. These results revealed that an altered expression of genes involved in one-carbon metabolism, including GNMT, PEMT, MTR, and CBS, may lead to the down-regulation of key gene(s) in HDL cholesterol assembly.

Consistent with the present study, several recent studies have reported the interaction between maternal and post-weaning diets in health outcomes in later life of offspring. Maternal folate depletion and offspring high-fat intake resulted in similar changes in hepatic gene expression with the largest change when these two effects were combined in adult offspring [39]. Offspring of dams fed a high-fat diet led to the severe response to a post-weaning methionine-choline deficient diet, resulting in steatosis, inflammation, and fibrosis in the liver [40]. Similarly, maternal protein restriction may program hypertension and hepatic steatosis in offspring, which were aggravated by a post-weaning high-fat diet [41]. These findings from animal studies demonstrate that the individual susceptibility to the development of liver diseases in later-life could be driven by maternal nutrition.

## 5. Conclusions

Maternal SPI diet is associated with more severe liver injury, altered homocysteine and one-carbon metabolism, as well as impaired HDL cholesterol assembly in response to postnatal ethanol exposure in offspring. These results suggest that altered hepatic gene expression and liver development observed at postnatal day 21 may confer several detrimental risk factors to the offspring, which can be sustained into adulthood. Further studies are warranted to determine whether the altered regulation of one-carbon metabolism may contribute to the higher susceptibility to other types of liver injury in offspring of dams fed an SPI diet.

## Figures and Tables

**Table 1:** Effects of maternal diet on body and organ weights in in ethanol-fed rat offspring.

	Diet (Maternal/Offspring)				Two-Way ANOVA
	CAS/CON	CAS/EtOH	SPI/CON	SPI/EtOH	
Body weight (g)					
3 weeks	62.1 ± 1.0 ^a^	64.6 ± 1.5 ^a^	54.3 ± 2.1 ^b^	55.1 ± 1.8 ^b^	M
8 weeks	400.1 ± 6.1	400.1 ± 4.8	385.6 ± 10.1	383.1 ± 10.2	
15 weeks(at sacrifice)	533.4 ± 12.3	497.9 ± 10.8	531.8 ± 22.8	511.6 ± 20.0	
Organ weight (g)					
Liver	15.0 ± 0.4	16.9 ± 0.8	15.3 ± 1.3	17.0 ± 1.3	
Epididymal fat	14.0 ± 1.3	10.7 ± 0.7	12.7 ± 1.1	10.4 ± 1.6	E
Relative liver weight (g/100 g body weight)					
Liver	2.8 ± 0.1 ^b^	3.4 ± 0.1 ^a^	2.9 ± 0.1 ^b^	3.3 ± 0.1 ^a^	E
Epididymal fat	2.6 ± 0.2 ^a^	2.1 ± 0.1 ^a,b^	2.4 ± 0.1 ^a,b^	2.0 ± 0.2 ^b^	E

Data are expressed as means ± SEM (*n* = 7–8). Significant effects of maternal diet (M), offspring ethanol diet (E), and their interaction (M × E) were analyzed by two-way ANOVA (*p* < 0.05). Means without a common superscript significantly differ based on post-hoc analysis (*p* < 0.05). Data that were not normally distributed were log-transformed before the statistical analysis. CAS, casein; SPI, low-isoflavone soy protein isolate; CON, control; EtOH, ethanol.

**Table 2:** Correlation between serum GPT level and hepatic expressions of genes related to one-carbon metabolism.

Hepatic Gene Expression (mRNA)	Correlation Coefficient (*r*)	*p*-Value
GNMT	−0.341	0.141
PEMT	−0.457	0.043
BHMT	−0.599	0.005
MTR	−0.690	0.001
CBS	−0.631	0.003

Correlation between two variables was analyzed by Pearson correlation coefficient (*n* = 20). Data that were not normally distributed were log-transformed before the statistical analysis. GNMT, Glycine *N*-methyltransferase; PEMT, Phosphatidylethanolamine *N*-methyltransferase; BHMT, Betaine-homocysteine *S*-methyltransferase; MTR, 5-Methyltetrahydrofolate-homocysteine methyltransferase; CBS, Cystathionine beta-synthase.

**Table 3:** Correlation between serum GPT level and hepatic expressions of genes related to cholesterol metabolism.

Hepatic Gene Expression (mRNA)	Correlation Coefficient (*r*)	*p*-Value
ABCA1	–0.594	0.006
ABCG5	–0.544	0.016
LCAT	–0.754	< 0.001
LDLR	–0.697	0.001
LXRα	–0.631	0.003
SRB1	–0.542	0.020

Correlation between two variables was analyzed by Pearson correlation coefficient (*n* = 18‒20). Data that were not normally distributed were log-transformed before the statistical analysis.

## Supplementary Materials

The following are available online at http://www.mdpi.com/2072-6643/10/3/332/s1, Table S1: Primer sequences for real-time PCR analysis (SYBR green), Table S2: Primer sequences for real-time PCR analysis (Taqman).
