# Supplementary material for: Maternal Consumption of Low-Isoflavone Soy Protein Isolate Confers the Increased Predisposition to Alcoholic Liver Injury in Adult Rat Offspring
Source: Nutrients. 2018 Mar 10;10(3):332. doi: 10.3390/nu10030332 (PMC5872750; doi:10.3390/nu10030332)
Supplement: Supplementary file 1 [file nutrients-10-00332-s001.docx]

**Supplemental Table S1**. Primer sequences for real-time PCR analysis (SYBR green)

| Gene | Forward (5′- 3′) | Reverse (5′- 3′) |
| --- | --- | --- |
| APOA1 | GGCAGAGACTATGTGTCCCAGTTT | TTGAACCCAGAGTGTCCCAGTT |
| CBS | TGCATTATCGTGATGCCTGAGAA | GGGAATCGAATCTGGCGTTG |
| LXRα | GAACAGATCCGCTTGAAGAA | ATGGCCAGCTCAGTAAAGTG |
| MCP1 | TAGCATCCACGTGCTGTCTC | CCGACTCATTGGGATCATCT |
| MTR | ACTTGCGCAAACTCCGCTATG | TGCCAAGGATTCTGTCAACCTG |
| TNFα | CCCATTACTCTGACCCCTTT | TGAGCATCGTAGTTGTTGGA |
| ACTB | CACACTGTGCCCATCTATGA | CCGATAGTGATGACCTGACC |

APOA1, apolipoprotein A1; CBS, cystathionine beta-synthase; LXRα, liver X receptor alpha; MCP1, monocyte chemoattractant protein 1; MTR, 5-methyltetrahydrofolate-homocysteine methyltransferase; TNFα, tumor necrosis factor alpha; ACTB, actin, beta

**Supplemental Table S2**. Primer sequences for real-time PCR analysis (Taqman)

| Gene | Product number | Gene | Product number |
| --- | --- | --- | --- |
| ABCA1 | Rn00710172_m1 | HMGCR | Rn00565598_m1 |
| ABCG5 | Rn00587092_m1 | LCAT | Rn00500505_m1 |
| APOA2 | Rn00565403_m1 | LDLR | Rn00598442_m1 |
| BHMT | Rn00578255_m1 | PEMT | Rn00564517_m1 |
| CHOP | Rn01458526_m1 | SRB1 | Rn00580588_m1 |
| CYP7A1 | Rn00564065_m1 | ACTB | Rn00667869_m1 |
| GNMT | Rn00567215_m1 |  |  |

ABCA1, ATP-binding cassette, subfamily A, member 1; ABCG5, ATP-binding cassette, subfamily G (WHITE), member 5; APOA2, apo-lipoprotein A2; BHMT, betaine-homocysteine methyltransferase; CHOP, DNA-damage inducible transcript 3; CYP7A1, cholesterol 7α hydroxylase; GNMT, glycine N-methyltransferase; HMGCR, 3-hydroxy-3-methylglutaryl-Coenzyme A reductase; LCAT, lecithin cholesterol acyltransferase; LDLR, low density lipoprotein receptor; PEMT, phosphatidylethanolamine N-methyltransferase; SRB1, scavenger receptor class B, member 1; ACTB, actin, beta
